# Supplementary material for: Multi‐dataset identification of innovative feature genes and molecular mechanisms in keratoconus
Source: J Cell Mol Med. 2024 Sep 19;28(18):e70079. doi: 10.1111/jcmm.70079 (PMC11412914; doi:10.1111/jcmm.70079)
Supplement: Supplementary file 2 — Material S2. [file JCMM-28-e70079-s002.zip › 7.GSEA analysis/1.ARL11/index.html]

Index for xtools.gsea.Gsea my\_analysis.Gsea.1678852737554

### GSEA Report for Dataset ARL11

#### Enrichment in phenotype: **h (8 samples)**

- 71 / 177 gene sets are upregulated in phenotype **h**- 0 gene sets are significant at FDR < 25%- 1 gene sets are significantly enriched at nominal pvalue < 1%- 2 gene sets are significantly enriched at nominal pvalue < 5%- Snapshot of enrichment results- Detailed enrichment results in html format- Detailed enrichment results in TSV format (tab delimited text)- Guide to interpret results

#### Enrichment in phenotype: **l (8 samples)**

- 106 / 177 gene sets are upregulated in phenotype **l**- 0 gene sets are significantly enriched at FDR < 25%- 0 gene sets are significantly enriched at nominal pvalue < 1%- 0 gene sets are significantly enriched at nominal pvalue < 5%- Snapshot of enrichment results- Detailed enrichment results in html format- Detailed enrichment results in TSV format (tab delimited text)- Guide to interpret results

#### Dataset details

- The dataset has 34729 features (genes)- No probe set => gene symbol collapsing was requested, so all 34729 features were used

#### Gene set details

- Gene set size filters (min=15, max=500) resulted in filtering out 9 / 186 gene sets- The remaining 177 gene sets were used in the analysis- List of gene sets used and their sizes (restricted to features in the specified dataset)

#### Gene markers for the **h** *versus* **l** comparison

- The dataset has 34729 features (genes)- # of markers for phenotype **h**: 16532 (47.6% ) with correlation area 48.4%- # of markers for phenotype **l**: 18197 (52.4% ) with correlation area 51.6%- Detailed rank ordered gene list for all features in the dataset- Heat map and gene list correlation  profile for all features in the dataset- Butterfly plot of significant genes

#### Global statistics and plots

- Plot of p-values *vs.* NES- Global ES histogram

#### Other

- Parameters used for this analysis

#### Comments

- Timestamp used as random seed: 1678852737554

#### Citing GSEA and MSigDB

To cite your use of the GSEA software please reference the following:

- Subramanian, A., Tamayo, P., et al. (2005, PNAS). - Mootha, V. K., Lindgren, C. M., et al. (2003, Nature Genetics).

For use of the Molecular Signatures Database (MSigDB), to cite please reference   
one or more of the following as appropriate, along with the source for the gene set as listed on the gene set page:

- Liberzon A, et al. (Bioinformatics, 2011). - Liberzon A, et al. (Cell Systems 2015).

---

Report: my\_analysis.Gsea.1678852737554.rpt   by user: EDY

xtools.gsea.Gsea [Wed, Mar 15, '23 11 AM 58]

Website: www.gsea-msigdb.org/gsea
Questions & Suggestions: Contact page
